# 3-(4-Bromo­phen­yl)-1-phenyl-1*H*-pyrazole-4-carbaldehyde

**DOI:** 10.1107/S1600536812046752

**Published:** 2012-11-24

**Authors:** Mahmoud Elkady, Peter R. W. E. F. Keck, Dieter Schollmeyer, Stefan Laufer

**Affiliations:** a Eberhard-Karls-University Tübingen, Auf der Morgenstelle 8, 72076 Tübingen, Germany; b University Mainz, Institut of Organic Chemistry, Duesbergweg 10-14, 55099 Mainz, Germany

## Abstract

The asymmetric unit of the title compound, C_16_H_11_BrN_2_O, contains two independent mol­ecules with slightly different geometries. The 4-bromo­benzene ring forms dihedral angles of 26.0 (2) and 39.9 (7)° with the pyrazole ring in the two mol­ecules while the phenyl ring is oriented at 19.7 (5) and 7.3 (0)° with respect to the pyrazole ring.

## Related literature

For the biological activity of inhibitors for the microsomal prostaglandin E_2_ synthase-1 (mPGES-1) and 5-lipoxygenase (5-LO), see: Elkady *et al.* (2012 ▶). For details of the synthesis, see: Rathelot *et al.* (2002 ▶).
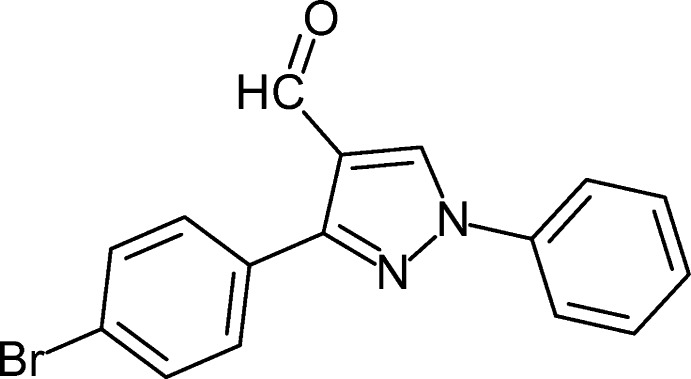


## Experimental

### 

#### Crystal data

C_16_H_11_BrN_2_O
*M* *_r_* = 327.18
Triclinic, 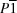

*a* = 9.6716 (8) Å
*b* = 11.4617 (9) Å
*c* = 13.8257 (10) Å
α = 113.497 (5)°
β = 92.753 (6)°
γ = 93.753 (6)°
*V* = 1397.91 (19) Å^3^
*Z* = 4
Mo *K*α radiation
μ = 2.94 mm^−1^
*T* = 298 K
0.34 × 0.18 × 0.06 mm

#### Data collection

Stoe IPDS 2T diffractometer
Absorption correction: multi-scan (*MULABS*; Blessing, 1995 ▶) *T*_min_ = 0.477, *T*_max_ = 0.660
14312 measured reflections
6740 independent reflections
2856 reflections with *I* > 2σ(*I*)
*R*_int_ = 0.063

#### Refinement

*R*[*F* ^2^ > 2σ(*F* ^2^)] = 0.052
*wR*(*F* ^2^) = 0.158
*S* = 0.92
6740 reflections
361 parameters
H-atom parameters constrained
Δρ_max_ = 0.52 e Å^−3^
Δρ_min_ = −0.73 e Å^−3^

### 

Data collection: *X-AREA* (Stoe & Cie, 2010 ▶); cell refinement: *X-AREA*; data reduction: *X-RED* (Stoe & Cie, 2010 ▶); program(s) used to solve structure: *SIR97* (Altomare *et al.*, 1999 ▶); program(s) used to refine structure: *SHELXL97* (Sheldrick, 2008 ▶); molecular graphics: *PLATON* (Spek, 2009 ▶); software used to prepare material for publication: *PLATON*.

## Supplementary Material

Crystal structure: contains datablock(s) I, global. DOI: 10.1107/S1600536812046752/bt6856sup1.cif

Structure factors: contains datablock(s) I. DOI: 10.1107/S1600536812046752/bt6856Isup2.hkl

Supplementary material file. DOI: 10.1107/S1600536812046752/bt6856Isup3.cml

Additional supplementary materials:  crystallographic information; 3D view; checkCIF report

## Figures and Tables

**Fig. 1.**
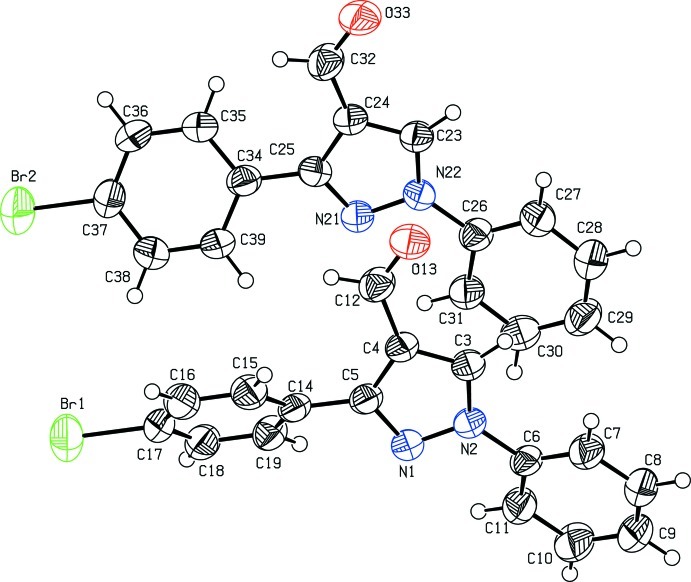
View of compound I. Displacement ellipsoids are drawn at the 50% probability level.

## supplementary crystallographic information

### Comment

We synthesized and evaluated inhibitors for the microsomal prostaglandin E_2_ synthase-1 (mPGES-1) and 5-lipoxygenase (5-LO) (Elkady *et al.*, 2012). The title compound was synthesized to obtain a template which leads to series of different derivates of the pyrazole scaffold (Rathelot *et al.*, 2002) the asymmetric unit of the crystal structure contains two slightly different molecules.

The 4-Bromobenzene ring is oriented with dihedral angles of 26.0 (2)°, 39.9 (7)° (first and second molecule) with the pyrazole ring. The phenyl ring is oriented with dihedral angles of 19.7 (5)°, 7.3 (0)° with the pyrazole ring.

### Experimental

The compound was prepared by a Vilsmeyer–Haack reaction. Phosphoryl chloride (4.16 ml, 44.7 mmol) was added dropwise to ice-cooled solution of 7 ml dimethylformamide. The mixture was left stirring for 30 min at 273 K. 4.3 g (14.9 mmol) of (*E*)-1-(1-(4-bromophenyl)ethylidene)-2-phenylhydrazine were dissolved in 10 ml dimethylformamide, and then slowly added to the mixture. The mixture was heated to 343 K and left for stirring for 4 h. The mixture was cooled to 273 K, quenched by water and adjusted to pH 12 with aqueous saturated sodium carbonate solution. The product was extracted with ethyl acetate three times, dried over anhydrous sodium sulfate and finally concentrated under vacuum. The product was purified by washing with methanol. Crystals of the title compound were obtained by slow evaporation of methanol at room temperature.

### Refinement

Hydrogen atoms were placed at calculated positions with C—H = 0.95 Å (aromatic) or 0.99–1.00 Å (*sp*^3^ C-atom). All H atoms were refined with isotropic displacement parameters (set at 1.2–1.5 times of the *U*_eq_ of the parent atom).

### Crystal data

**Table:**

C_16_H_11_BrN_2_O	*Z* = 4
*M**_r_* = 327.18	*F*(000) = 656
Triclinic, *P*1	*D*_x_ = 1.555 Mg m^−^^3^
Hall symbol: -P 1	Mo *K*α radiation, λ = 0.71073 Å
*a* = 9.6716 (8) Å	Cell parameters from 5561 reflections
*b* = 11.4617 (9) Å	θ = 2.7–28.0°
*c* = 13.8257 (10) Å	µ = 2.94 mm^−^^1^
α = 113.497 (5)°	*T* = 298 K
β = 92.753 (6)°	Block, colourless
γ = 93.753 (6)°	0.34 × 0.18 × 0.06 mm
*V* = 1397.91 (19) Å^3^	

### Data collection

**Table:**

Stoe IPDS 2T diffractometer	6740 independent reflections
Radiation source: sealed X-ray tube, 12 x 0.4 mm long-fine focus	2856 reflections with *I* > 2σ(*I*)
Graphite monochromator	*R*_int_ = 0.063
Detector resolution: 6.67 pixels mm^-1^	θ_max_ = 28.0°, θ_min_ = 2.6°
rotation method scans	*h* = −12→11
Absorption correction: multi-scan (*MULABS*; Blessing, 1995)	*k* = −15→15
*T*_min_ = 0.477, *T*_max_ = 0.660	*l* = −18→18
14312 measured reflections	

### Refinement

**Table:**

Refinement on *F*^2^	Primary atom site location: structure-invariant direct methods
Least-squares matrix: full	Secondary atom site location: difference Fourier map
*R*[*F*^2^ > 2σ(*F*^2^)] = 0.052	Hydrogen site location: inferred from neighbouring sites
*wR*(*F*^2^) = 0.158	H-atom parameters constrained
*S* = 0.92	*w* = 1/[σ^2^(*F*_o_^2^) + (0.0707*P*)^2^] where *P* = (*F*_o_^2^ + 2*F*_c_^2^)/3
6740 reflections	(Δ/σ)_max_ < 0.001
361 parameters	Δρ_max_ = 0.52 e Å^−^^3^
0 restraints	Δρ_min_ = −0.73 e Å^−^^3^

### Special details

**Table:**

Geometry. All e.s.d.'s (except the e.s.d. in the dihedral angle between two l.s. planes) are estimated using the full covariance matrix. The cell e.s.d.'s are taken into account individually in the estimation of e.s.d.'s in distances, angles and torsion angles; correlations between e.s.d.'s in cell parameters are only used when they are defined by crystal symmetry. An approximate (isotropic) treatment of cell e.s.d.'s is used for estimating e.s.d.'s involving l.s. planes.
Refinement. Refinement of *F*^2^ against ALL reflections. The weighted *R*-factor *wR* and goodness of fit *S* are based on *F*^2^, conventional *R*-factors *R* are based on *F*, with *F* set to zero for negative *F*^2^. The threshold expression of *F*^2^ > σ(*F*^2^) is used only for calculating *R*-factors(gt) *etc*. and is not relevant to the choice of reflections for refinement. *R*-factors based on *F*^2^ are statistically about twice as large as those based on *F*, and *R*- factors based on ALL data will be even larger.

### Fractional atomic coordinates and isotropic or equivalent isotropic displacement parameters (Å^2^)

**Table:**

	*x*	*y*	*z*	*U*_iso_*/*U*_eq_	
Br1	0.32448 (7)	0.17418 (5)	−0.10411 (4)	0.0974 (3)	
N1	0.2200 (3)	0.6242 (3)	0.3849 (2)	0.0482 (8)	
N2	0.2418 (3)	0.7380 (3)	0.4705 (2)	0.0456 (7)	
C3	0.3427 (4)	0.8149 (3)	0.4591 (3)	0.0468 (9)	
H3	0.3743	0.8967	0.5078	0.056*	
C4	0.3921 (4)	0.7507 (3)	0.3612 (3)	0.0456 (9)	
C5	0.3115 (4)	0.6312 (3)	0.3181 (3)	0.0457 (9)	
C6	0.1618 (4)	0.7599 (3)	0.5587 (3)	0.0450 (9)	
C7	0.1515 (4)	0.8810 (4)	0.6313 (3)	0.0570 (10)	
H7	0.1947	0.9507	0.6226	0.068*	
C8	0.0763 (5)	0.8990 (4)	0.7178 (3)	0.0660 (12)	
H8	0.0710	0.9814	0.7683	0.079*	
C9	0.0099 (5)	0.7980 (4)	0.7302 (4)	0.0692 (12)	
H9	−0.0417	0.8110	0.7881	0.083*	
C10	0.0202 (5)	0.6772 (4)	0.6565 (4)	0.0703 (13)	
H10	−0.0256	0.6078	0.6641	0.084*	
C11	0.0976 (4)	0.6567 (4)	0.5708 (3)	0.0579 (11)	
H11	0.1061	0.5741	0.5219	0.070*	
C12	0.5114 (4)	0.7921 (4)	0.3201 (3)	0.0537 (10)	
H12	0.5384	0.7351	0.2559	0.064*	
O13	0.5781 (3)	0.8928 (3)	0.3614 (2)	0.0668 (8)	
C14	0.3153 (4)	0.5230 (4)	0.2171 (3)	0.0456 (9)	
C15	0.3337 (4)	0.5387 (4)	0.1243 (3)	0.0556 (10)	
H15	0.3454	0.6209	0.1264	0.067*	
C16	0.3352 (4)	0.4363 (4)	0.0292 (3)	0.0626 (11)	
H16	0.3460	0.4488	−0.0326	0.075*	
C17	0.3207 (4)	0.3157 (4)	0.0265 (3)	0.0610 (11)	
C18	0.3015 (4)	0.2953 (4)	0.1167 (3)	0.0583 (11)	
H18	0.2904	0.2128	0.1139	0.070*	
C19	0.2991 (4)	0.3994 (4)	0.2112 (3)	0.0524 (10)	
H19	0.2863	0.3863	0.2725	0.063*	
Br2	0.83318 (7)	0.15393 (6)	−0.08996 (5)	0.1091 (3)	
N21	0.7249 (3)	0.6207 (3)	0.3891 (2)	0.0473 (8)	
N22	0.7455 (3)	0.7353 (3)	0.4734 (2)	0.0453 (7)	
C23	0.8472 (4)	0.8114 (3)	0.4605 (3)	0.0487 (9)	
H23	0.8795	0.8934	0.5084	0.058*	
C24	0.8949 (4)	0.7456 (3)	0.3631 (3)	0.0478 (9)	
C25	0.8145 (4)	0.6255 (3)	0.3209 (3)	0.0457 (9)	
C26	0.6588 (4)	0.7623 (4)	0.5591 (3)	0.0484 (9)	
C27	0.6652 (5)	0.8807 (4)	0.6382 (3)	0.0638 (12)	
H27	0.7294	0.9451	0.6390	0.077*	
C28	0.5759 (5)	0.9050 (4)	0.7174 (4)	0.0692 (12)	
H28	0.5813	0.9858	0.7721	0.083*	
C29	0.4796 (5)	0.8119 (4)	0.7167 (3)	0.0623 (11)	
H29	0.4192	0.8288	0.7700	0.075*	
C30	0.4738 (4)	0.6930 (4)	0.6357 (4)	0.0620 (11)	
H30	0.4084	0.6289	0.6342	0.074*	
C31	0.5632 (4)	0.6669 (4)	0.5567 (3)	0.0553 (10)	
H31	0.5589	0.5858	0.5024	0.066*	
C32	0.9995 (4)	0.7970 (4)	0.3158 (3)	0.0593 (11)	
H32	1.0124	0.7504	0.2449	0.071*	
O33	1.0705 (3)	0.8950 (3)	0.3615 (3)	0.0702 (9)	
C34	0.8187 (4)	0.5151 (3)	0.2220 (3)	0.0477 (9)	
C35	0.9388 (4)	0.4886 (4)	0.1687 (3)	0.0595 (11)	
H35	1.0188	0.5443	0.1966	0.071*	
C36	0.9428 (4)	0.3834 (4)	0.0769 (4)	0.0657 (12)	
H36	1.0239	0.3691	0.0420	0.079*	
C37	0.8276 (5)	0.2994 (4)	0.0365 (3)	0.0630 (11)	
C38	0.7060 (5)	0.3220 (4)	0.0872 (3)	0.0623 (11)	
H38	0.6271	0.2647	0.0595	0.075*	
C39	0.7026 (4)	0.4291 (4)	0.1782 (3)	0.0543 (10)	
H39	0.6202	0.4446	0.2114	0.065*	

### Atomic displacement parameters (Å^2^)

**Table:**

	*U*^11^	*U*^22^	*U*^33^	*U*^12^	*U*^13^	*U*^23^
Br1	0.1105 (5)	0.0877 (4)	0.0613 (3)	−0.0126 (3)	0.0273 (3)	−0.0034 (3)
N1	0.0435 (19)	0.0514 (17)	0.0455 (17)	−0.0017 (14)	0.0070 (14)	0.0157 (15)
N2	0.0432 (19)	0.0482 (17)	0.0444 (17)	−0.0026 (14)	0.0089 (14)	0.0180 (15)
C3	0.045 (2)	0.0484 (19)	0.047 (2)	−0.0034 (17)	0.0084 (17)	0.0208 (18)
C4	0.040 (2)	0.051 (2)	0.046 (2)	−0.0008 (16)	0.0083 (17)	0.0208 (18)
C5	0.038 (2)	0.055 (2)	0.045 (2)	0.0029 (17)	0.0066 (17)	0.0212 (18)
C6	0.041 (2)	0.055 (2)	0.041 (2)	0.0006 (17)	0.0086 (16)	0.0219 (18)
C7	0.059 (3)	0.054 (2)	0.057 (2)	−0.0005 (19)	0.015 (2)	0.021 (2)
C8	0.065 (3)	0.063 (3)	0.058 (3)	0.004 (2)	0.018 (2)	0.010 (2)
C9	0.068 (3)	0.080 (3)	0.054 (3)	−0.002 (2)	0.023 (2)	0.021 (2)
C10	0.076 (3)	0.073 (3)	0.066 (3)	−0.007 (2)	0.024 (2)	0.033 (3)
C11	0.062 (3)	0.055 (2)	0.054 (2)	0.002 (2)	0.015 (2)	0.018 (2)
C12	0.053 (3)	0.062 (2)	0.052 (2)	0.003 (2)	0.0122 (19)	0.028 (2)
O13	0.0653 (19)	0.0557 (16)	0.075 (2)	−0.0154 (14)	0.0153 (16)	0.0234 (15)
C14	0.034 (2)	0.060 (2)	0.043 (2)	0.0019 (17)	0.0079 (16)	0.0206 (18)
C15	0.057 (3)	0.059 (2)	0.053 (2)	−0.0051 (19)	0.0065 (19)	0.026 (2)
C16	0.062 (3)	0.077 (3)	0.046 (2)	−0.011 (2)	0.006 (2)	0.024 (2)
C17	0.053 (3)	0.068 (3)	0.047 (2)	−0.006 (2)	0.0162 (19)	0.007 (2)
C18	0.055 (3)	0.050 (2)	0.064 (3)	0.0037 (18)	0.016 (2)	0.016 (2)
C19	0.052 (2)	0.056 (2)	0.051 (2)	0.0059 (18)	0.0121 (18)	0.0232 (19)
Br2	0.1057 (5)	0.0904 (4)	0.0834 (4)	−0.0047 (3)	0.0324 (4)	−0.0156 (3)
N21	0.0460 (19)	0.0457 (16)	0.0471 (18)	−0.0029 (14)	0.0079 (15)	0.0162 (15)
N22	0.0445 (19)	0.0414 (15)	0.0457 (17)	−0.0028 (13)	0.0053 (14)	0.0138 (14)
C23	0.046 (2)	0.0447 (19)	0.052 (2)	−0.0031 (17)	0.0096 (18)	0.0164 (18)
C24	0.043 (2)	0.049 (2)	0.051 (2)	−0.0016 (17)	0.0074 (18)	0.0198 (18)
C25	0.040 (2)	0.049 (2)	0.046 (2)	−0.0013 (16)	0.0015 (17)	0.0177 (18)
C26	0.047 (2)	0.052 (2)	0.048 (2)	0.0028 (17)	0.0075 (18)	0.0221 (19)
C27	0.063 (3)	0.055 (2)	0.061 (3)	−0.007 (2)	0.012 (2)	0.012 (2)
C28	0.069 (3)	0.067 (3)	0.059 (3)	0.006 (2)	0.017 (2)	0.010 (2)
C29	0.056 (3)	0.082 (3)	0.055 (3)	0.011 (2)	0.017 (2)	0.031 (2)
C30	0.058 (3)	0.066 (3)	0.066 (3)	−0.004 (2)	0.013 (2)	0.031 (2)
C31	0.056 (3)	0.052 (2)	0.055 (2)	−0.0034 (19)	0.011 (2)	0.0189 (19)
C32	0.064 (3)	0.053 (2)	0.062 (3)	0.001 (2)	0.020 (2)	0.023 (2)
O33	0.0640 (19)	0.0537 (16)	0.088 (2)	−0.0133 (14)	0.0199 (16)	0.0247 (16)
C34	0.041 (2)	0.055 (2)	0.049 (2)	0.0007 (17)	0.0062 (17)	0.0231 (19)
C35	0.040 (2)	0.063 (2)	0.062 (3)	−0.0044 (18)	0.0065 (19)	0.012 (2)
C36	0.043 (2)	0.072 (3)	0.070 (3)	0.004 (2)	0.020 (2)	0.015 (2)
C37	0.066 (3)	0.060 (2)	0.052 (2)	0.004 (2)	0.018 (2)	0.009 (2)
C38	0.053 (3)	0.064 (2)	0.057 (3)	−0.011 (2)	0.006 (2)	0.013 (2)
C39	0.043 (2)	0.062 (2)	0.052 (2)	−0.0064 (18)	0.0100 (18)	0.018 (2)

### Geometric parameters (Å, º)

**Table:**

Br1—C17	1.890 (4)	Br2—C37	1.880 (4)
N1—C5	1.330 (4)	N21—C25	1.326 (4)
N1—N2	1.363 (4)	N21—N22	1.358 (4)
N2—C3	1.330 (4)	N22—C23	1.337 (4)
N2—C6	1.421 (4)	N22—C26	1.428 (5)
C3—C4	1.383 (5)	C23—C24	1.375 (5)
C3—H3	0.9300	C23—H23	0.9300
C4—C5	1.416 (5)	C24—C25	1.420 (5)
C4—C12	1.445 (5)	C24—C32	1.446 (5)
C5—C14	1.457 (5)	C25—C34	1.451 (5)
C6—C7	1.364 (5)	C26—C27	1.357 (6)
C6—C11	1.372 (5)	C26—C31	1.374 (5)
C7—C8	1.382 (6)	C27—C28	1.379 (6)
C7—H7	0.9300	C27—H27	0.9300
C8—C9	1.362 (6)	C28—C29	1.366 (6)
C8—H8	0.9300	C28—H28	0.9300
C9—C10	1.367 (6)	C29—C30	1.371 (6)
C9—H9	0.9300	C29—H29	0.9300
C10—C11	1.381 (5)	C30—C31	1.377 (5)
C10—H10	0.9300	C30—H30	0.9300
C11—H11	0.9300	C31—H31	0.9300
C12—O13	1.190 (4)	C32—O33	1.193 (5)
C12—H12	0.9300	C32—H32	0.9300
C14—C15	1.383 (5)	C34—C39	1.383 (5)
C14—C19	1.384 (5)	C34—C35	1.391 (5)
C15—C16	1.371 (6)	C35—C36	1.363 (6)
C15—H15	0.9300	C35—H35	0.9300
C16—C17	1.365 (6)	C36—C37	1.361 (6)
C16—H16	0.9300	C36—H36	0.9300
C17—C18	1.377 (6)	C37—C38	1.384 (6)
C18—C19	1.376 (5)	C38—C39	1.368 (5)
C18—H18	0.9300	C38—H38	0.9300
C19—H19	0.9300	C39—H39	0.9300
			
C5—N1—N2	105.3 (3)	C25—N21—N22	106.1 (3)
C3—N2—N1	112.2 (3)	C23—N22—N21	111.8 (3)
C3—N2—C6	128.4 (3)	C23—N22—C26	128.6 (3)
N1—N2—C6	119.4 (3)	N21—N22—C26	119.6 (3)
N2—C3—C4	107.3 (3)	N22—C23—C24	107.1 (3)
N2—C3—H3	126.4	N22—C23—H23	126.5
C4—C3—H3	126.4	C24—C23—H23	126.5
C3—C4—C5	104.7 (3)	C23—C24—C25	105.3 (3)
C3—C4—C12	126.5 (3)	C23—C24—C32	124.8 (3)
C5—C4—C12	128.3 (3)	C25—C24—C32	129.8 (3)
N1—C5—C4	110.5 (3)	N21—C25—C24	109.7 (3)
N1—C5—C14	119.1 (3)	N21—C25—C34	119.2 (3)
C4—C5—C14	130.3 (3)	C24—C25—C34	131.1 (3)
C7—C6—C11	120.5 (3)	C27—C26—C31	120.5 (4)
C7—C6—N2	120.7 (3)	C27—C26—N22	121.2 (3)
C11—C6—N2	118.8 (3)	C31—C26—N22	118.2 (3)
C6—C7—C8	119.4 (4)	C26—C27—C28	119.7 (4)
C6—C7—H7	120.3	C26—C27—H27	120.2
C8—C7—H7	120.3	C28—C27—H27	120.2
C9—C8—C7	120.9 (4)	C29—C28—C27	120.9 (4)
C9—C8—H8	119.5	C29—C28—H28	119.5
C7—C8—H8	119.5	C27—C28—H28	119.5
C8—C9—C10	119.1 (4)	C28—C29—C30	118.7 (4)
C8—C9—H9	120.4	C28—C29—H29	120.6
C10—C9—H9	120.4	C30—C29—H29	120.6
C9—C10—C11	120.9 (4)	C29—C30—C31	121.0 (4)
C9—C10—H10	119.5	C29—C30—H30	119.5
C11—C10—H10	119.5	C31—C30—H30	119.5
C6—C11—C10	119.1 (4)	C26—C31—C30	119.1 (4)
C6—C11—H11	120.4	C26—C31—H31	120.4
C10—C11—H11	120.4	C30—C31—H31	120.4
O13—C12—C4	125.6 (4)	O33—C32—C24	124.6 (4)
O13—C12—H12	117.2	O33—C32—H32	117.7
C4—C12—H12	117.2	C24—C32—H32	117.7
C15—C14—C19	117.7 (4)	C39—C34—C35	117.1 (4)
C15—C14—C5	122.1 (3)	C39—C34—C25	120.7 (3)
C19—C14—C5	120.3 (3)	C35—C34—C25	122.2 (3)
C16—C15—C14	121.7 (4)	C36—C35—C34	122.0 (4)
C16—C15—H15	119.2	C36—C35—H35	119.0
C14—C15—H15	119.2	C34—C35—H35	119.0
C17—C16—C15	119.1 (4)	C37—C36—C35	119.7 (4)
C17—C16—H16	120.5	C37—C36—H36	120.2
C15—C16—H16	120.5	C35—C36—H36	120.2
C16—C17—C18	121.3 (4)	C36—C37—C38	120.2 (4)
C16—C17—Br1	119.3 (3)	C36—C37—Br2	119.7 (3)
C18—C17—Br1	119.4 (3)	C38—C37—Br2	120.1 (3)
C19—C18—C17	118.7 (4)	C39—C38—C37	119.5 (4)
C19—C18—H18	120.6	C39—C38—H38	120.2
C17—C18—H18	120.6	C37—C38—H38	120.2
C18—C19—C14	121.5 (4)	C38—C39—C34	121.5 (4)
C18—C19—H19	119.2	C38—C39—H39	119.2
C14—C19—H19	119.2	C34—C39—H39	119.2
			
C5—N1—N2—C3	0.2 (4)	C25—N21—N22—C23	−0.8 (4)
C5—N1—N2—C6	−178.3 (3)	C25—N21—N22—C26	176.9 (3)
N1—N2—C3—C4	−0.0 (4)	N21—N22—C23—C24	0.8 (4)
C6—N2—C3—C4	178.3 (4)	C26—N22—C23—C24	−176.6 (4)
N2—C3—C4—C5	−0.1 (4)	N22—C23—C24—C25	−0.5 (4)
N2—C3—C4—C12	−173.0 (4)	N22—C23—C24—C32	176.1 (4)
N2—N1—C5—C4	−0.3 (4)	N22—N21—C25—C24	0.5 (4)
N2—N1—C5—C14	180.0 (3)	N22—N21—C25—C34	179.3 (3)
C3—C4—C5—N1	0.3 (4)	C23—C24—C25—N21	−0.0 (5)
C12—C4—C5—N1	172.9 (4)	C32—C24—C25—N21	−176.4 (4)
C3—C4—C5—C14	180.0 (4)	C23—C24—C25—C34	−178.6 (4)
C12—C4—C5—C14	−7.4 (7)	C32—C24—C25—C34	5.0 (7)
C3—N2—C6—C7	20.3 (6)	C23—N22—C26—C27	5.3 (6)
N1—N2—C6—C7	−161.4 (4)	N21—N22—C26—C27	−171.9 (4)
C3—N2—C6—C11	−158.2 (4)	C23—N22—C26—C31	−178.0 (4)
N1—N2—C6—C11	20.0 (5)	N21—N22—C26—C31	4.7 (5)
C11—C6—C7—C8	0.4 (7)	C31—C26—C27—C28	0.7 (7)
N2—C6—C7—C8	−178.1 (4)	N22—C26—C27—C28	177.3 (4)
C6—C7—C8—C9	−1.5 (7)	C26—C27—C28—C29	−0.9 (8)
C7—C8—C9—C10	1.0 (8)	C27—C28—C29—C30	0.4 (7)
C8—C9—C10—C11	0.6 (8)	C28—C29—C30—C31	0.3 (7)
C7—C6—C11—C10	1.1 (7)	C27—C26—C31—C30	−0.0 (7)
N2—C6—C11—C10	179.7 (4)	N22—C26—C31—C30	−176.7 (4)
C9—C10—C11—C6	−1.7 (7)	C29—C30—C31—C26	−0.5 (7)
C3—C4—C12—O13	−6.5 (7)	C23—C24—C32—O33	11.3 (7)
C5—C4—C12—O13	−177.6 (4)	C25—C24—C32—O33	−172.9 (4)
N1—C5—C14—C15	139.7 (4)	N21—C25—C34—C39	26.1 (6)
C4—C5—C14—C15	−40.0 (6)	C24—C25—C34—C39	−155.4 (4)
N1—C5—C14—C19	−39.7 (5)	N21—C25—C34—C35	−152.2 (4)
C4—C5—C14—C19	140.6 (4)	C24—C25—C34—C35	26.3 (7)
C19—C14—C15—C16	0.4 (6)	C39—C34—C35—C36	0.5 (6)
C5—C14—C15—C16	−179.0 (4)	C25—C34—C35—C36	178.8 (4)
C14—C15—C16—C17	−1.2 (7)	C34—C35—C36—C37	−1.6 (7)
C15—C16—C17—C18	1.5 (7)	C35—C36—C37—C38	1.4 (7)
C15—C16—C17—Br1	−179.4 (3)	C35—C36—C37—Br2	−179.8 (4)
C16—C17—C18—C19	−1.0 (7)	C36—C37—C38—C39	−0.2 (7)
Br1—C17—C18—C19	180.0 (3)	Br2—C37—C38—C39	−179.0 (3)
C17—C18—C19—C14	0.1 (6)	C37—C38—C39—C34	−1.0 (7)
C15—C14—C19—C18	0.2 (6)	C35—C34—C39—C38	0.8 (6)
C5—C14—C19—C18	179.6 (4)	C25—C34—C39—C38	−177.5 (4)
